# Supplementary material for: Mutations in PCYT2 disrupt etherlipid biosynthesis and cause a complex hereditary spastic paraplegia
Source: Brain. 2019 Oct 22;142(11):3382–97. doi: 10.1093/brain/awz291 (PMC6821184; doi:10.1093/brain/awz291)
Supplement: awz291_Supplementary_Data [file awz291_supplementary_data.zip › awz291-Suppl_data/Supplementary_Data4.zip!/Supplementary information 4 - plasma lipidomics.zip!/Plasma lipidomics/Control vs Patient3a/Statistical plots and data/Random forest.pdf]

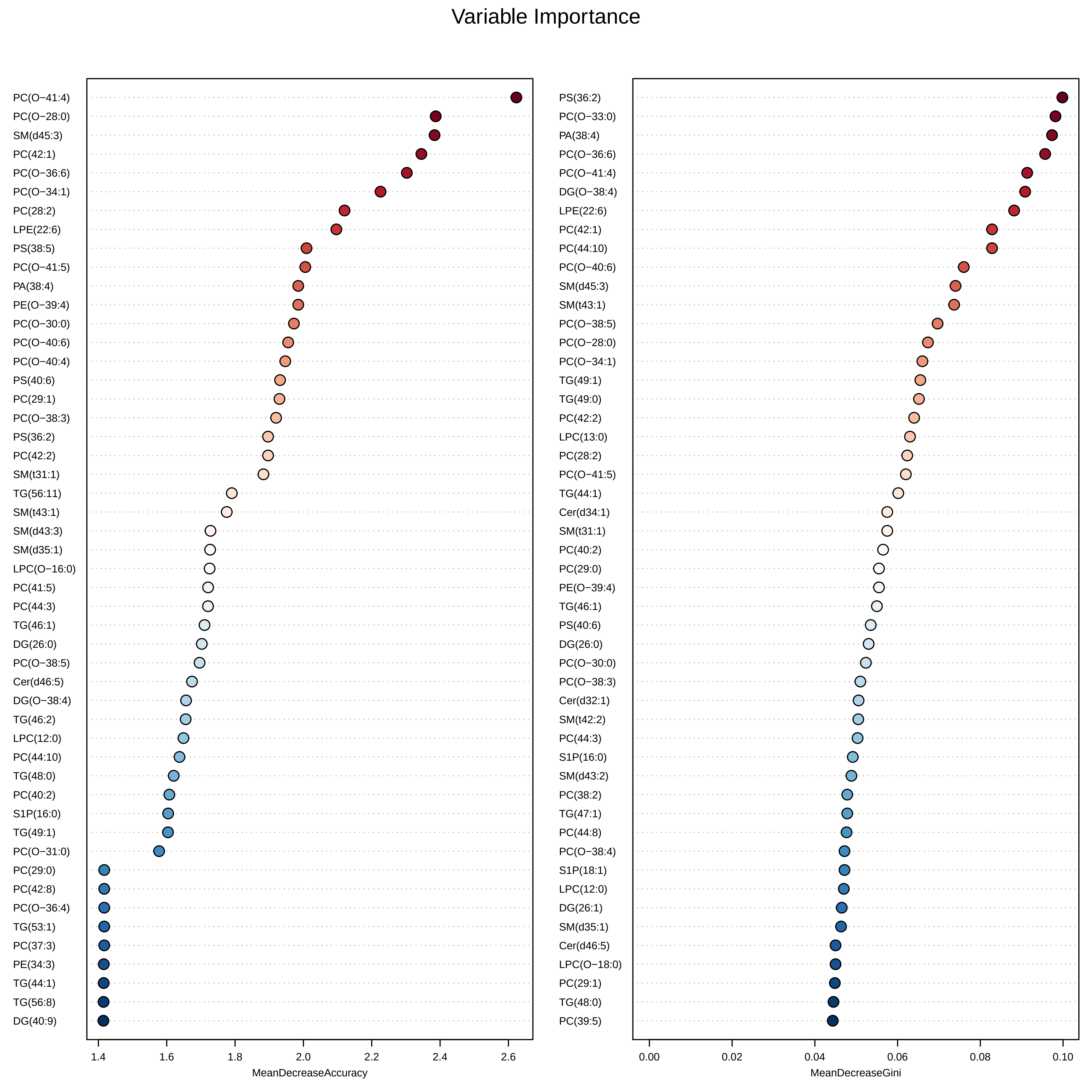

Variable Importance
PC(O−41:4)
PC(O−28:0)
SM(d45:3)
PC(42:1)
PC(O−36:6)
PC(O−34:1)
PC(28:2)
LPE(22:6)
PS(38:5)
PC(O−41:5)
PA(38:4)
PE(O−39:4)
PC(O−30:0)
PC(O−40:6)
PC(O−40:4)
PS(40:6)
PC(29:1)
PC(O−38:3)
PS(36:2)
PC(42:2)
SM(t31:1)
TG(56:11)
SM(t43:1)
SM(d43:3)
SM(d35:1)
LPC(O−16:0)
PC(41:5)
PC(44:3)
TG(46:1)
DG(26:0)
PC(O−38:5)
Cer(d46:5)
DG(O−38:4)
TG(46:2)
LPC(12:0)
PC(44:10)
TG(48:0)
PC(40:2)
S1P(16:0)
TG(49:1)
PC(O−31:0)
PC(29:0)
PC(42:8)
PC(O−36:4)
TG(53:1)
PC(37:3)
PE(34:3)
TG(44:1)
TG(56:8)
DG(40:9)
1.4
1.6
1.8
2.0
2.2
2.4
2.6
MeanDecreaseAccuracy
PS(36:2)
PC(O−33:0)
PA(38:4)
PC(O−36:6)
PC(O−41:4)
DG(O−38:4)
LPE(22:6)
PC(42:1)
PC(44:10)
PC(O−40:6)
SM(d45:3)
SM(t43:1)
PC(O−38:5)
PC(O−28:0)
PC(O−34:1)
TG(49:1)
TG(49:0)
PC(42:2)
LPC(13:0)
PC(28:2)
PC(O−41:5)
TG(44:1)
Cer(d34:1)
SM(t31:1)
PC(40:2)
PC(29:0)
PE(O−39:4)
TG(46:1)
PS(40:6)
DG(26:0)
PC(O−30:0)
PC(O−38:3)
Cer(d32:1)
SM(t42:2)
PC(44:3)
S1P(16:0)
SM(d43:2)
PC(38:2)
TG(47:1)
PC(44:8)
PC(O−38:4)
S1P(18:1)
LPC(12:0)
DG(26:1)
SM(d35:1)
Cer(d46:5)
LPC(O−18:0)
PC(29:1)
TG(48:0)
PC(39:5)
0.00
0.02
0.04
0.06
0.08
0.10
MeanDecreaseGini
